# 

**Published:** 1889-04-20

**Authors:** 


### THE STUDENTS' COLUMN.

### "A COMPLETE COMPENDIUM OF THE SCIENCE AND ART OF SURGERY." *

The author states, from twelve years' experience in reading with candidates for a surgical diploma, he found that owing to the huge size attained by most of the standard text-books, the knowledge obtained from their perusal is not well arranged in the student's mind, nor readily at command during the trying ordeal of an examination. Hence the compendium which is now presented. Every subject belonging to the domain of surgery is treated of, excepting diseases of the ear, dental surgery, and diseases of women, the excuse for the omission being "limited space." The treatment recommended is that which experience has demonstrated to be the most efficacious, and to this cause no possible objection can be offered. But in the case of operations the author has, as far as possible, followed literally the description of the surgeon who first performed or proposed each operation. But many operative procedures have been improved upon by successors in the field, and the author should have added that such improvements are also noticed. We have not space for long quotations from Dr. Dale's book, but in order to show the concise style we extract as follows:

"*Injuries to Arteries.*—Arteries may be bruised. The effects of the contusion may be transitory, but occasionally inflammation follows, leading to—1. Narrowing or obliteration of the vessel from contraction of its coats. 2. Plugging from the formation of a clot which produces cessation of the pulse below. 3. Ulceration and hæmorrhage.

As a book of the kind we can only praise the "Epitome of Surgery." We are not, however, clear—notwithstanding what the author says of the size of surgical works—that the "Epitome of Surgery" was required. Druit's "Vade Mecum," edited by Stanley Boyd, still holds its own. Bryant's "Practice of Surgery" is to the front, while "Clarke's Manual" does not lag far behind. It is quite possible to have too much of a good thing. There is no doubt that the new book will be useful, not only to the student, but also to the practising surgeon who may wish to refresh his memory; but the same may be said of various other works. At present the student is perplexed by the number of textbooks issued on all manner of subjects. It would certainly be desirable if some authority or other would indicate what books it is desirable for students to use. The General Medical Council of Registration and Education might do so. If the council took upon themselves this procedure, and recommended Dr. Dale's "Epitome of Surgery," we should have no objection to make. If, on the other hand, the council named some other work, we do not think Dr. Dale would have much cause for complaint.

* "Epitome of Surgery, being a Complete Compendium of the Science and Art of Surgery." By Ridley Dale, M.D., M.R.C.S., etc.; Member of the Northumberland and Durham Medical Society, Surgeon to the Hospital for Foreign Seamen, Sunderland. H. K. Lewis, 136, Gower-street, W.C. 1889.

### IMPORTANT NOTICE.

### A ROYAL NUMBER

Of The Hospital will be published on April 27th, 1889. It will contain an account of the Public Life and Work of their Royal Highnesses

THE PRINCE AND PRINCESS OF WALES.

*** The monthly part for May will contain Special Portraits of their Royal Highnesses. See notice below as to separate copies.

Intending Subscribers should send their names without delay to the Publisher, The Hospital, 139-140, Salisbury Court, Fleet Street, E.C.

### Scraps and Gleanings.

Sir Morell Mackenzie has gone on a voyage to Teneriffe.

The grounds at the back of Wigan Infirmary are being skilfully laid out.

The London Fever Hospital has received a cheque for £200 from Thomas Vernall, Esq.

The death is announced at Gibraltar of Mr. W. B. Rankin, the distinguished philanthropist.

Adelaide Hospital, Dublin, is in debt £3,000, and appeals to the public for further support.

The Royal Blind Asylum, Edinburgh, has received a donation of £400 from an anonymous friend.

Miss Gordon has given the library of the late General Gordon to Southampton Free Library.

Patients' registration fees at Birmingham Ear and Throat Hospital amounted last year to £392.

The mortality at Edinburgh for the week ending April 6th was 85, a death-rate of 17 in the 1,000.

Shrewsbury Dispensary reports an aggregate number of 20,527 members. This sounds well.

Five hundred collecting boxes are being sent to Liverpool workshops by the Hospital Saturday Fund.

The Government has resolved to institute an enquiry regarding the condition of London cemeteries.

Meath Hospital treated 1,269 in-patients, 2,532 out-patients, and 9,596 dispensary patients last year.

Ninety patients were treated in the Cork-street Fever Hospital, Dublin, during the month of March.

Dr. Van Lauer, who was for many years physician-in-ordinary to the late Emperor William, died on the 8th inst.

Mr. Arthur Oliver has been elected medical officer of Malvern Rural Hospital, in place of Dr. Dawson, resigned.

Sir John Lubbock will lay the foundation-stone of the Central Free Public Library at Battersea on Thursday, May 2nd.

The deaths in London last week were 1,572, being 251 below the average in the corresponding weeks of the last ten years.

Joseph Redmond, who murdered his wife lately at Dundee, has been certified to be insane, and has been removed to an asylum.

The three Medical Research Scholarships of the Grocer's Company, value £250 each, must be applied for before the end of April.

A dramatic performance was given on Thursday, the 18th, at the Lyric Hall, Hammersmith, in aid of the West London Hospital.

The festival dinner of the London Lock Hospital will be held at the Hotel Metropole on July 3rd. Lord Kinnaird will take the chair.

Through an accident that occurred on the 24th January in a silver mine in Bolivia, nineteen natives and three Englishmen were killed.

An errand-boy named Frampton, aged 13 years, died at Portsmouth last week from hydrophobia. He was bitten by a mad dog on November 17th.

Mr. John Bright has left no legacies to public institutions, it being his strong opinion that the support of such charities is the duty of the living.

Mr. F. M. Dean, of Deptford, is taking up the scheme of Convalescent Homes for Friendly Societies, which works so well in the Northern Counties.

A committee of the principal citizens of Cork has been formed to celebrate in 1890 the centenary of Father Matthew, the Apostle of Temperance.

Several cases of cholera are reported from Colombo, and owing to the prevalence of this disease the pearl fishery at the Bay of Cordately has been suspended.

The "Modern Man" described in last week's *Scot's Observer* is Sir Joseph Lister. A full and appreciative account is given of Sir Joseph's services to biology and surgery.

Mr. Moore, surgeon of Yiewsley, has died from diphtheria, contracted from sucking a tracheotomy tube inserted in his child's throat, who was also suffering from that disease.

There is great distress in Sicily. Very sad reports of the sufferings of the people come from Palermo, and at Modica the town distributes 2,500 rations of provisions daily.

Mr. Henry Tate has offered £1,000 to the building fund of the new hospital for women, on condition that the whole of the needed £19,000 is subscribed during the next four months.

Cholera has broken out at Surat, the unhappy town lately nearly destroyed by fire. Lord Reay, Governor of Bombay, has gone to Surat to take measures for the relief of the sufferers.

Amongst the candidates for the fellowship of the Royal Society are David Cunningham, whose researches regarding cholera are so well known, and Dr. G. R. Yeo, the eminent pathologist.

At a late discussion at Liverpool on the Trades Council and the Hospital Saturday Fund, it was stated that for every £1 contributed, 12s. is used in management and 8s. in the relief of suffering.

An influential committee, of which the Duke of Portland is chairman, has been formed to make arrangements for holding a *Bal Poudre* at the Hotel Metropole, on the 28th of May, in aid of the funds of the Home of Rest for Horses.

At Stockport, Grace Bennett, an elderly woman, having a cold, bought a bottle of cough mixture from a chemist, and took three parts of it during the night. Next morning she was dead, and a coroner's jury has found that she died from an overdose of morphia.

At Darlington, the police recently sold the furniture of a Mr. Glaister, of Hurworth, which was distrained for a vaccination fine. Mr. Glaister explained that the furniture was seized to defray fines under what he considered to be an unjust law, and asked the public not to bid for it. Those who had come to the sale obeyed this request, and the furniture was bought back by the owner.

### THE GUIDE TO STRETCHER AND BEARER COMPANY DRILL.

Staff-Sergeant W. Kay Waterson writes from the Volunteer Medical Staff Corps (No 4 Company), Birkbeck Institution, Bream's-buildings: May I ask space in your columns to correct an impression the review of this book in your issue of April 6 might possibly create, namely, that the drill set forth is a new system. The drill of the Medical Staff Corps is referred to as a "new drill." The system of drill set out in the "Guide" is, however, the existing drill used by the Medical Staff Corps, and is in accordance with the authorised Manual of 1885 (now out of print). It is not a new system advanced by the writer. The "Regulations for the Medical Department" (522 pp., 8vo., price 2s. 6d.) contain absolutely no directions as to drill. An attempt has been made to bring together into 18 pp. square the four [Part I.] such parts of the regulations as show the duties of those conveying a wounded man from the front to the home hospitals, and to make available the drill of the Manual of 1885 with a few alterations which have been already introduced at the Aldershot Training School.

### Amusements and Relaxation.

### ACROSTIC COMPETITION.

As so few competitors have entered for the above competition, we have decided to discontinue it until the autumn, and to run the Word Competition only.

### Word Competition.

Commenced April 6th, Ends June 29th.

Three Prizes of 15s., 10s., 5s., will be given for the largest number of words derived from the word set for dissection.

Proper names, abbreviations, foreign words, words of less than four letters, and repetitions are barred; plurals, and past and present participles of verbs, are allowed. Nuttall's *Standard* dictionary only to be used.

N.B.—All words sent in must be arranged *alphabetically*, with correct total affixed.

The word for dissection for this, the *Third*, week of the quarter being

"C A M B R I D G E."

### Results of First Weeks.

| Names. | Correct Totals. | Names. | Correct Totals. |
|---|---|---|---|
| Patience | 173 | C. J. S. | 134 |
| Nurse Duty | 172 | See-saw | 133 |
| Lightowlers | 172 | Rosemont | 131 |
| Scrooge | 172 | Jenny Wren | 126 |
| Reldas | 171 | Eric | 122 |
| Tinie | 170 | Tiny | 120 |
| K. G. | 169 | N'importe Qui | 117 |
| Lauriston | 165 | Green | 112 |
| Spes | 165 | W. G. R. | 109 |
| Gretta | 162 | Robe | 107 |
| Francesca | 158 | Beryl | 107 |
| Heatherbell | 149 | Nesta | 91 |
| Sycamore | 149 | Daffodil | 89 |
| Arctus | 145 | A Schoolgirl | 81 |
| Amicus | 144 | Sheeny | 78 |
| Scratcher | 143 | Alice R. | 63 |
| Jemima | 138 | | |

### Notice to all Correspondents.

N.B.—All letters referring to this page, which do not arrive at 139 and 140, Salisbury-court, London, E.C., by the *first post on Thursdays*, and are not addressed PRIZE EDITOR, will in future be disqualified and disregarded.

### Conditions.

I. Every paper sent in must be clearly written, and one side only must be used. All competitors must mark at the head of their papers the number of their competition.

II. Each paper must be signed by the author with his or her real name and address. A *nom de plume* may be added if the writer does not desire to be referred to by us by his real name. In the case of all prize-winners, however, the real name and address will be published.

III. All communications relating to prize competitions should be addressed to "The Prize Editor, The Hospital, 139 and 140, Salisbury-court, London, E.C." Competitors are requested not to include in letters, etc., to the Prize Editor communications on matters of other business. All letters must be fully prepaid.

IV. The Prize Editor of The Hospital is the sole judge of the competitions, and his decision is in all cases final and without appeal.

V. The Prize Editor reserves the right to publish any paper sent in, whether it receives a prize or not. He does not hold himself responsible for any MSS. sent, nor can he undertake to return rejected competitions.

